# ART outcomes of patients in women with Isolated Hypogonadotropic Hypogonadism: a retrospective study in China

**DOI:** 10.1186/s12884-023-05579-5

**Published:** 2023-04-14

**Authors:** Wanxue Xu, Rong li, Jie Qiao

**Affiliations:** 1 Center for Reproductive Medicine, Department of Obstetrics and Gynecology, Peking University Third Hospital, Beijing, 100191 China; 2 Key Laboratory of Assisted Reproduction, Ministry of Education, Beijing, 100191 China; 3 Beijing Key Laboratory of Reproductive Endocrinology and Assisted Reproduction, Beijing, 100191 China

**Keywords:** Isolated Hypogonadotropic Hypogonadism, Infertility, FOI, Clinical pregnancy, Ovarian response

## Abstract

**Background:**

Isolated Hypogonadotropic Hypogonadism (IHH) is a rare reproductive disorder caused by the dysfunction of the gonadotropin-releasing hormone axis. Patients with IHH typically fail to enter or develop through puberty and retain infertile without an exogenous hormone supplement. This study aimed to investigate the population characteristics and reproductive outcomes in IHH patients undergoing assisted reproductive technology (ART) treatment, and evaluate the best-performed predictor for ovarian response and clinical pregnancy in patients with IHH.

**Methods:**

This retrospective cohort study included 83 women with IHH who underwent fresh ART cycles and non-diagnosed controls (*n* = 676). The receiver operating characteristic curves were generated to assess the predictor for the ovarian response. Logistic regression analyses were performed to investigate the independent factors for clinical pregnancy in IHH.

**Results:**

The basal hormone levels were significantly lower in the IHH group compared to the control group. The fertilization rate and 2PN rate were significantly higher in IHH groups, as was the number of transferable embryos. The study identified that AMH was the best predictor of high ovarian response in IHH, with an AUC of 0.767 (0.573, 0.961). Conversely, the follicle-to-oocyte index (FOI) exhibited the highest AUC of 0.814 (0.642, 0.985) for predicting low ovarian response. Based on FOI values, the IHH patients were divided into two groups, and the study found a significant increase in clinical pregnancy rate (43.8%, 58%; *P* < 0.001) and live birth rate (37.5%, 58%; *P* < 0.001) from the low FOI to the normal FOI groups. Moreover, the number of oocytes retrieved, fertilized embryos/rate, 2PN embryos/rate, and number of excellent quality embryos were significantly higher in the normal FOI group (*P* < 0.001 or *P* = 0.005) than in the low FOI group. Logistic regression analyses revealed FOI to be the independent factor affecting clinical pregnancy in IHH patients.

**Conclusions:**

The study findings suggest that patients with IHH were good responders to IVF treatment. Although AMH was the best-performed predictor for the high ovarian response, FOI had the best capability in predicting the low ovarian response. FOI was an independent factor affecting clinical pregnancy in IHH undergoing IVF/ICSI.

**Supplementary Information:**

The online version contains supplementary material available at 10.1186/s12884-023-05579-5.

## Introduction

Idiopathic hypogonadotropic hypogonadism (IHH, MIM147950) is a rare genetic disease, occurring in 1:29,000 males and 1:125,000 females [1]. The hallmark of IHH is absent pubertal development, hypogonadotropism and infertility, which are attributed to disrupted gonadotropin-releasing hormone (GnRH) secretion and/or action. This disruption prevents the onset of puberty and leads to low sex steroid levels and hypogonadotropic levels of luteinizing hormone (LH) and follicle-stimulating hormone (FSH). Consequently, patients with IHH remain infertile and require hormone supplementation to conceive naturally. The use of exogenous gonadotropins (Gn) for ovarian stimulation in women with IHH is a suitable and acceptable approach to mimic the normal menstrual cycle and restore ovarian function. In clinical practice, the reproductive outcome for inducing ovulation in IHH patients is promising with guided coitus or intrauterine insemination (IUI). However, for IHH patients with induced ovulation failure and who also harbored other causes for infertility (male factor and fallopian tubes factor), assisted reproductive technology (ART) becomes a valuable option to achieve patient fertility desires. Nevertheless, limited research has been studied on the reproductive characteristics and ART outcomes in IHH due to the rarity of the disease.

Obtaining optimal ART outcomes and minimizing risk for hyper-stimulation (ovarian hyper-stimulation syndrome, OHSS) remains challenging for clinical practice. Variability exists in the ovarian response to comparable doses of ovarian stimulation among women. Thus, accurately predicting the responses is vital to achieving a satisfying reproductive outcome. Several ovarian biomarkers, such as anti-Müllerian hormone (AMH), basal FSH, and antral follicle count (AFC), have been gradually suggested in predicting ovarian response and adjusting ovarian stimulation. However, these predictors have certain clinical practice limitations [2–4]. Alviggi group initially proposed to use follicle-to-oocyte index (FOI) to identify ovarian responsiveness in reflecting the dynamic nature of follicular growth in response to Gn stimulation [5]. The FOI is defined as the ratio between the number of oocytes retrieved at the end of ovarian stimulation and the number of AFC at the beginning of stimulation [5]. Thus, FOI is considered a qualitative biomarker for assessing the extent to which ovarian reserve is utilized based on the actual number of oocytes obtained during the stimulation cycle [5]. FOI may help identify low prognosis patients undergoing in vitro fertilization (IVF) treatment [6]. Despite limited research, the association between FOI and IVF outcomes remains unclear. To date, predictors for ovarian response in IHH patients and the association between FOI and IVF pregnancy outcomes have not been fully explored.

This retrospective study aimed to describe the population characteristics and reproductive outcomes in IHH patients, identify the best predictors for ovarian response and evaluate the potential of FOI to predict clinical pregnancy in IHH patients undergoing ART. The ultimate goal of this study is to optimize ovarian stimulation medication and improve ART outcomes in patients with IHH.

## Materials and methods

The studies involving human participants were reviewed and approved by the Ethics Committee of Peking University Third Hospital. This study did not require informed consent for participation by the national legislation and institutional requirements.

### Patients

**Fig. 1:**
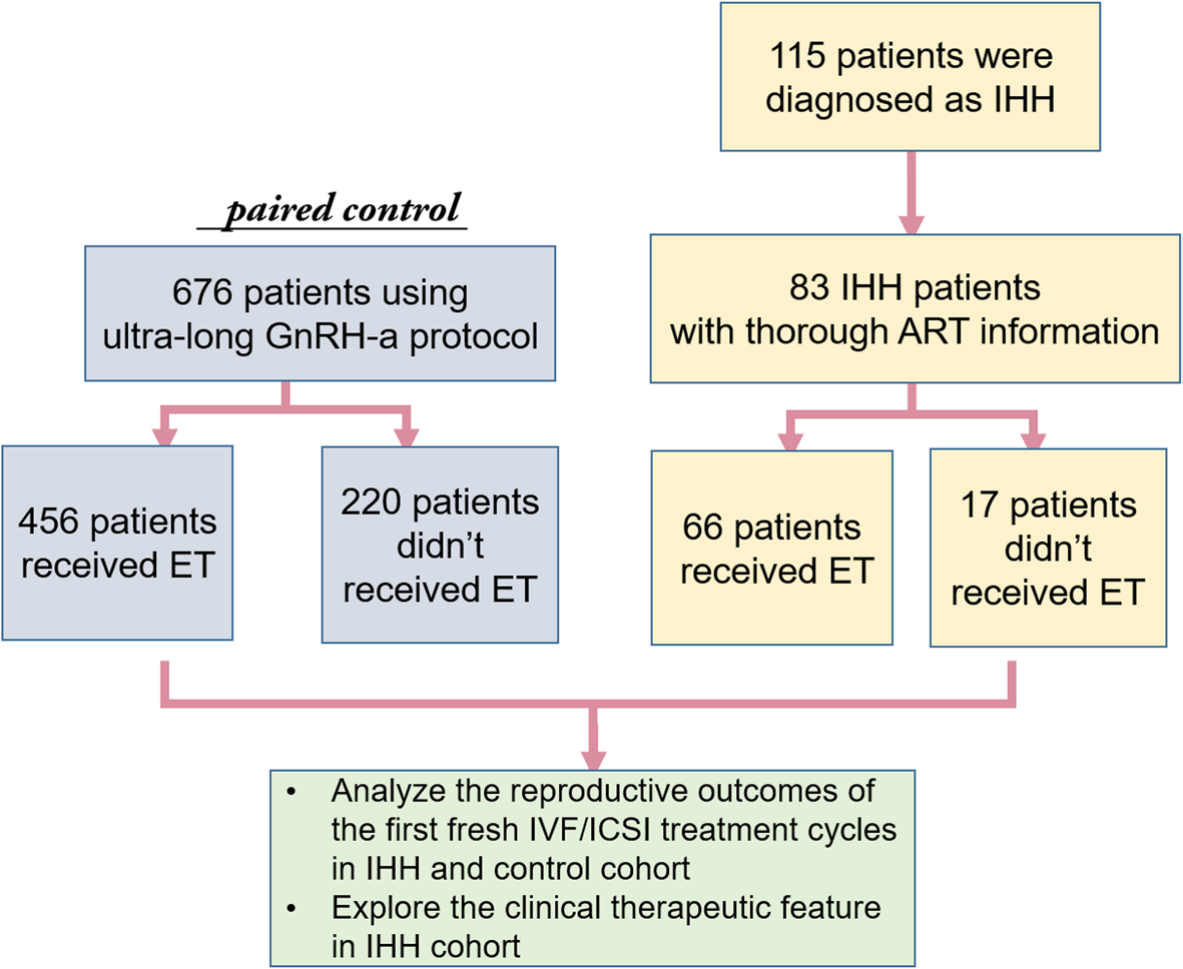
Enrollment of IHH and Study strategy. ET: embryo transfer; GnRH-a: gonadotropin-releasing hormone analogs

This retrospective cohort study included 115 women diagnosed with IHH who underwent oocyte retrieval at the Reproductive Center of Peking University Third Hospital between January 2010 and October 2021. The definition of IHH used in this study was consistent with previous publications [7, 8]. In brief, IHH was defined as: i) absent or incomplete sexual maturation; ii) serum estradiol < 73.4 pmol/L in the presence of low or normal levels of serum gonadotropins; iii) no functional cause for hypogonadism. Among the 115 women with IHH, 83 patients with detailed clinical information regarding IVF cycles were included in this study. The major cause for IVF was repeated failure to conceive after multiple attempts of ovulation induction with or without intrauterine insemination (IUI). Intracytoplasmic sperm injection (ICSI) was performed for 13 of them with coexisting male factor infertility. The inclusion criteria for the 676 patients in the control group were as follows: i) infertility due to male factors, tubal factor or idiopathic causes (excluding endometriosis, adenomyosis and polycystic ovarian syndrome); ii) use of ultra-long gonadotropin-releasing hormone agonist (GnRH-a) protocol; and iii) matching background characteristics with the IHH group (Fig. 1).

### Controlled Ovarian Stimulation (COS) protocols

In IHH patients, prior to the super ovulation, more than two cycles of hormone replacement therapy (HRT) were performed to provide endometrial regeneration. Then they received daily urinary gonadotropin for injection (HMG, Livzon, China) with or without recombinant human FSH for injection (rFSH, Gonal-F, Merck Serono, Germany). The ultra-long GnRH agonist (GnRH-a) protocol was performed in the control group. Initially, Triptorelin Acetate (Diphereline, Ipsen, France) was administered in either the early- follicular phase or mid-luteal phase of the last menstrual period, with a dose of 1.8 mg or 3.75 mg. After 28 or 35 days, hormone tests and ultrasound were monitored to determine pituitary suppression, following which rFSH was administered.

When at least two dominant follicles (with a diameter ≥ 18 mm) were developed, all patients were triggered by either 10,000 IU human chorionic gonadotropin (HCG, Livzon, China) or 250 ug recombinant HCG for injection (rHCG, Ovidral, Merck Serono, Germany). Thirty-six hours later, the transvaginal ultrasound-guided oocyte retrieval was performed. On the same day, IVF or ICSI was performed based on the evaluation of semen analysis. The embryo transfer (ET) was performed with either two embryos or one blastocyst after 3 or 5 days of oocyte retrieval. Clinical pregnancy was defined as a gestational sac in which a fetal heartbeat is detected by ultrasound 28 to 30 days after embryo transfer.

### Cycle outcomes

Cycle characteristics, including total gonadotropins dosage, starting gonadotropins dosage, estrogen levels on the day of HCG trigger, and endometrial thickness on the day of HCG trigger, were documented. In addition, cycle outcomes were examined, including the number of oocytes retrieved, the number of two pronuclei (2PN) fertilized oocytes, the number of transferred embryos, the method of fertilization, and causes for cycle cancellation. On the transfer day, embryo grading was assessed. Cleavage-stage embryos were evaluated according to the criteria of the Istanbul Embryo Evaluation Symposium [9] and blastocysts were evaluated using the Gardner grading system [10]. Fertilization rate and 2PN rate were calculated as following formulas:

$$\mathrm{IVF}\;\mathrm{fertilization}\;\mathrm{rate}\;=\;(2\mathrm{PN}\;+\;1\mathrm{PN}\;+\;\mathrm{multiple}\;\mathrm{PN})\;/\;\mathrm{number}\;\mathrm{of}\;\mathrm{oocytes}\;\mathrm{retrieved}$$

$$\mathrm{ICSI}\;\mathrm{fertilization}\;\mathrm{rate}\;=\;(2\mathrm{PN}\;+\;1\mathrm{PN}\;+\;\mathrm{multiple}\;\mathrm{PN})\;/\;\mathrm{number}\;\mathrm{of}\;\mathrm{MII}\;\mathrm{eggs}$$

$$2\mathrm{PN}\;\mathrm{rate}\;\mathrm{in}\;\mathrm{IVF}=\;2\mathrm{PN}\;/\;\mathrm{number}\;\mathrm{of}\;\mathrm{oocytes}\;\mathrm{retrieved}$$

$$2\mathrm{PN}\;\mathrm{rate}\;\mathrm{in}\;\mathrm{ICSI}=\;2\mathrm{PN}\;/\;\mathrm{number}\;\mathrm{of}\;\mathrm{MII}\;\mathrm{eggs}$$

### Follicle-to-oocyte index (FOI) definition

FOI was assessed as the ratio between the number of oocytes retrieved and the number of AFC at the beginning of stimulation per patient. In this study, FOI values were divided into two groups: low ovarian sensitivity (FOI values ≤ 0.50) and normal ovarian sensitivity (FOI > 0.5). The data of FOI in the tables are expressed by the median (25% quantile, 75% quantile) and compared per group.

### Ovarian response

Meet any two of the following criteria was classified into the high ovarian response group:

- 1) total number of follicles ≥ 15; 2) E2 on HCG trigger day ≥ 12,000 pmol/L; 3) history of OHSS/ previous risk of OHSS for miscarriage. Criteria for low ovarian response were based on 2016 POSEIDON criteria [11].

### Data analysis

Statistical analysis was conducted using GraphPad Prism 9.4.0 and SPSS26.0. The mean ± SD was used to present normally distributed data, while median ± quartile (25% quantile, 75% quantile) was used to present non-normally distributed data. Two different statistical tests were employed when analyzing group comparisons, depending on the distribution of the variables. For normally distributed variables, variance analysis and two-independent sample tests were conducted, while non-parametric Mann–Whitney U tests were used for non-normally distributed variables. For categorical variables, Fisher's exact test or Chi-square test was conducted as appropriate. The *P*-value < 0.05 was considered significant. To identify markers for predicting ovarian response in IHH, receiver operating characteristic (ROC) curves were generated. Univariate logistic regression analyses were carried out to assess variables which may be associated with clinical pregnancy in IHH. Multivariate logistic regression analyses were performed to estimate the associations between FOI and clinical pregnancy in IHH.

## Results

### All COS cycles

Demographic and basal characteristics and ART outcomes for 83 women in the IHH group and 676 women in the control group are shown in Table 1. The age, BMI, and partner's age were comparable between both groups. The basal hormones and AMH levels were significantly lower in the IHH group, while no differences were observed in the AFC between groups. The cycle characteristics, including starting Gn doses, E2 level on HCG day and endometrial thickness, were similar in both IHH group and controls. However, the IHH group required more days of ovarian stimulation and a larger total Gn dose than the control group. The number of oocytes retrieved per cycle (11 versus 12; *P* = 0.539) and FOI value for ovarian sensitivity (0.89 versus 1.03; *P* = 0.12) were similar between groups. Notably, fertilization rate and 2PN rate in both fertilized methods (IVF and ICSI) were significantly higher in the IHH group than in the control group, as were the number of excellent quality embryos and transferable embryos. The ET rate was also higher in the IHH group compared to the controls (*P* = 0.027). The most common cause of cycle cancellation was prevention for the ovarian hyper-stimulation syndrome (OHSS), which did not differ between groups. The clinical pregnancy rate (CPR) and live birth rate (LBR) were not significantly different between groups. However, among patients diagnosed with clinical pregnancy, a higher live birth rate was observed in the IHH groups (*P* = 0.02) with only one spontaneous loss of fetus at six weeks of gestation.

**Table 1:** Baseline characteristics and cycle outcomes in IHH and controls

**Baseline characteristics**	IHH group (*n* = 83)	Controls (*n* = 676)	*P* value
Age (years)	30 (28, 32)	31 (29, 32)	0.16
Partners Age (years)	31 (26,35)	32 (30, 34)	0.23
BMI (kg/m2)	21.4 (19.5, 24.0)	21 (19.9 22.1)	0.10
Basal FSH (mIU/mL)	1.36 (0.28, 3.69)	7.05 (5.95, 8.41)	< 0.001
Basal E2 (pmol/L)	108 (75.4, 146)	135 (98, 173)	< 0.01
Basal PRL (ng/mL)	6.74 (4.71, 10.60)	12.35 (9.01, 17.11)	< 0.001
Basal LH (mIU/mL)	0.32 (0.15, 1.25)	3.9 (2.66, 5.28)	< 0.001
Basal A (nmol/L)	4.8 (2.98, 7.01)	5.71 (4.45, 7.95)	< 0.01
AMH (ng/ml)	2.04 (1.24, 3.77)	2.42 (1.49, 4.04)	0.021
AFC	8 (5, 13)	9 (6, 12)	0.57
**Cycle characteristics and outcomes**			
Starting Gn doses (IU)	225 (150, 300)	225 (150, 300)	0.46
Stimulation days	13 (11, 15)	12 (11, 14	< 0.001
Total Gn doses (IU)	3412.5 (2400, 4200)	3075 (2325, 3900	< 0.001
Endometrial thickness (mm)	0.6 (0.5, 0.7)	11 (10, 12)	0.36
E2 level on HCG day (pmol/L)	7191 (4616, 11,561)	6755 (4629, 11,073.25)	0.26
Number of oocytes retrieved	11 (7.5, 15)	12 (8, 17)	0.54
FOI	0.89 (0.61, 1.15)	1.03 (0.91, 1.12)	0.12
Fertilization method (IVF/ ICSI)	69/12	593/82	0.49
Number of fertilized embryos	9 (6, 12)	8.5 (6, 12)	0.68
Number of 2PN embryos	7 (5, 10)	7 (4, 10)	0.37
Fertilization rate (%)			
IVF	81	76	< 0.001
ICSI	82	72	0.031
2PN rate (%)			
IVF	66.7	59.5	< 0.001
ICSI	76.9	64.1	0.018
Number of excellent quality embryos	6.5 (5.5, 10.5)	5 (3.75, 8)	< 0.001
Number of transferable embryos	4 (2,8)	3 (2,6)	0.024
**Embryo transferred (ET) outcomes**			
ET rate (%, n / N)	79.5 (66/83)	67.5 (456/676)	0.027
Number of transferred embryos			0.6
1 (%, n / N)	4.5 (3/66)	7.0 (32/456)	
2 (%, n / N)	95.5 (63/66)	93.0 (424/456)	
Cause for cancellation of ET (%)			
High risk for OHSS	76.5	58.2	0.19
No qualified embryos	11.8	17.3	0.74
Healthy factor (fever, abdominal pain etc.)	5.9	14.5	0.48
Personal factor	5.9	10	0.99
Clinical pregnancy rate (%)	55.5	59.9	0.41
Live birth rate (%)	53	49.6	0.59

The data are expressed by the median (25% quantile, 75% quantile), and the comparison between the two groups is performed by Mann Whitney test

*BMI* Body mass index, *FSH* Follicle stimulating hormone, *LH* Luteinizing hormone, *E2* Estradiol, *A* Androstenedione, *PRL* Prolactin, *Gn* Gonadotropins, *AFC* Antral follicle count, *FOI* Follicle-to-oocyte index

### Predictive markers in ovarian response in IHH

**Fig. 2:**
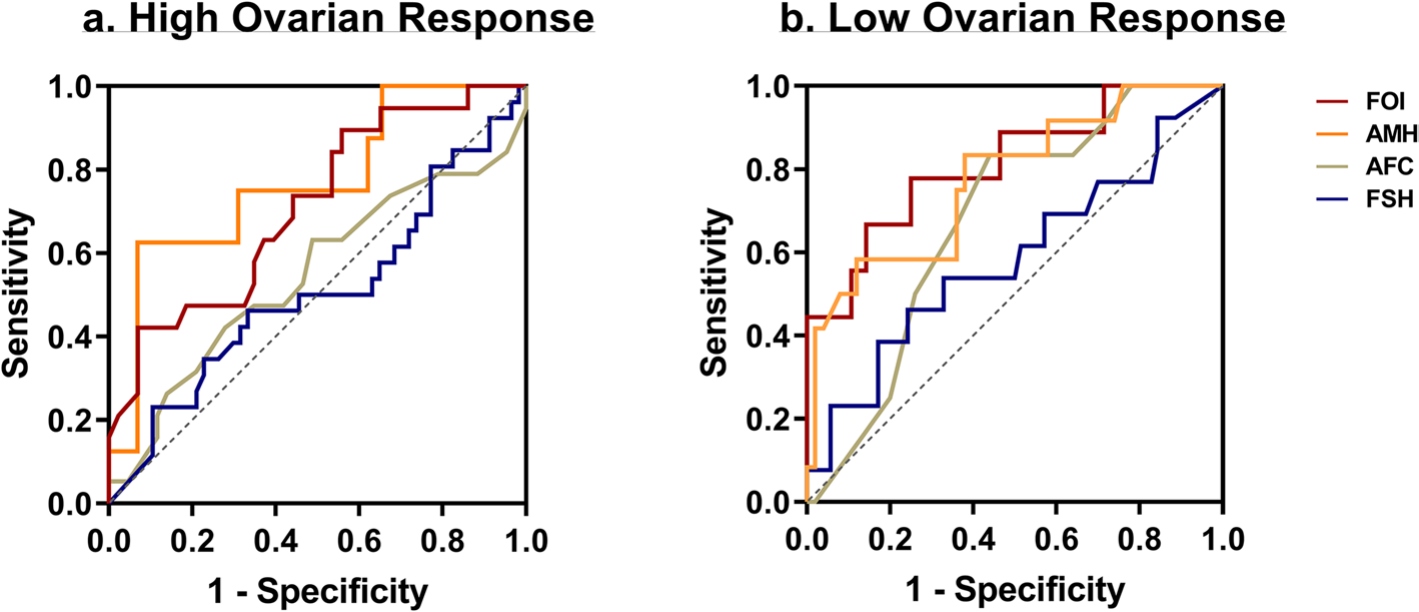
ROC curves of various markers to predict high ovarian response (**a**) and low ovarian response (**b**) in COS cycles

ROC curves were employed to evaluate the predictive performance of various factors for ovarian response (Fig. 2). AMH and FOI were found to have satisfactory AUC values of over 0.7 for predicting high and low ovarian response groups (*P* < 0.01 or *P* < 0.05) (Supplementary Table 1). Specifically, AMH exhibited the highest AUC [0.767 (0.573, 0.962)] for predicting high ovarian response (Fig. 2a), while FOI demonstrated the highest AUC [0.814 (0.642, 0.985)] for predicting low ovarian response (Fig. 2b). To further explore the ovarian sensitivity in IHH, patients were categorized into two groups based on the FOI value: low FOI (*n* = 20) with FOI value ≤ 0.5, and normal FOI (*n* = 63) with FOI > 0.5. The baseline characteristics did not differ significantly between the groups. However, the AMH, number of oocytes retrieved, fertilized embryos/rate, 2PN embryos/rate and number of excellent quality embryos were significantly higher in normal FOI group (*P* < 0.001 or *P* = 0.005) (Table 2). Moreover, the total Gn doses administered were significantly lower in the normal FOI group (*P* < 0.01). The clinical pregnancy rate and live birth rate significantly increased from the low FOI to the normal FOI groups (*P* < 0.001).

**Table 2:** Baseline characteristics and ovarian stimulation data in different follicle-to-oocyte index group in IHH cohort

**FOI group**	**Low FOI (*n* = 20)**	**Normal FOI (*n* = 63)**	***P* value**
Age (years)	32 (30, 34.5)	30 (28, 32)	0.34
Partners Age (years)	31.5 (30.2, 36.2)	32 (28.7, 33.2)	0.54
BMI (kg/m2)	23.0 (21.3, 24.6)	21.4 (20.1, 24.2)	0.76
Basal FSH (mIU/mL)	1.6 (0.18, 4.33)	1.70 (0.38, 3.83)	0.18
Basal E2 (pmol/L)	111.5 (95.75, 148)	108.5 (81.37, 152.75)	0.66
Basal PRL (ng/mL)	11.2 (7.9, 19.6)	8.29 (5.1, 10.6)	0.74
Basal LH (mIU/mL)	0.26 (0.2, 0.47)	0.39 (0.15, 1.58)	0.19
Basal A (nmol/L)	3.26 (2.99, 4.71)	4.88 (2.98, 7.06)	0.55
AMH (ng/ml)	0.97 (0.65, 2.4)	2.2 (1.61, 3.93)	< 0.001
AFC	12.5 (6.5, 17)	7.5 (5, 13)	< 0.001
Oocytes retrieved	5 (3, 6)	9 (7.7, 13.2)	< 0.001
FOI	0.39 (0.36, 0.52)	0.93 (0.74, 1.21)	< 0.001
Fertilized embryos	4.5 (2.5, 6.75)	9 (7, 13)	< 0.001
2PN embryos	4 (2.5, 5)	8 (6, 11.25)	< 0.001
Fertilization rate (%)			
IVF	80	85.7	< 0.001
ICSI	100	81.7	< 0.001
2PN rate (%)			
IVF	66.7	69.2	0.005
ICSI	100	79.3	< 0.001
Starting Gn doses (IU)	225 (225, 300)	225 (168.75, 300)	0.31
Stimulation days	14.5 (11.25, 15)	13 (11.75, 15)	0.22
Total Gn doses (IU)	3937.5 (2634.375, 5175)	3450 (2550, 4200)	< 0.01
E2 on HCG day (pmol/L)	5246 (3822.75, 7867)	7736.5 (5065.25, 12,729.5)	< 0.001
Excellent quality embryos	3 (1.25, 3.75)	6 (3.75, 8)	< 0.001
Transferable embryos	2.5 (2, 3)	4 (3, 9)	< 0.001
Transferred embryos	2 (1.75, 2)	2 (2, 2)	0.98
CPR (%)	7 (43.8%)	29 (58%)	< 0.001
LBR (%)	6 (37.5%)	29 (58%)	< 0.001

The data are expressed by the median (25% quantile, 75% quantile), and the comparison between the two groups is performed by Mann Whitney test and Pearson Chi square test

*BMI* Body mass index, *FSH* Follicle stimulating hormone, *LH* Luteinizing hormone, *E2* Estradiol, *A* Androstenedione, *PRL* Prolactin, *Gn* Gonadotropins, *AFC* Antral follicle count, *FOI* Follicle-to-oocyte index, *HCG* Human chorionic gonadotropin, *PN* Pronuclear, *CPR* Clinical pregnancy rate, *LBR* Live birth rate

### Clinical pregnancy in IHH

In order to further determine the primary factors affecting clinical pregnancy in women with IHH, a total of 66 out of 83 women in the IHH group who underwent ET were analyzed (Table 3). There was no difference in baseline characteristics between the pregnant and non-pregnant groups, but the AFC was higher in the pregnant group. The FOI was significantly higher in the pregnant group (*P* < 0.001), as were the number of oocytes retrieved, fertilized embryos (*P* < 0.001), 2PN embryos (*P* = 0.031), the number of excellent quality embryos (*P* < *0.001*) and transferable embryos (*P* = *0.022*) (Table 3).

**Table 3:** Baseline characteristics and cycle outcomes in clinical pregnancy and non-pregnancy women with IHH

	**Clinical pregnant**		***P* value**
	**Yes (*n* = 36)**	**No (*n* = 30)**	
Age (years)	30 (28, 31)	31 (28.5, 32.5)	0.20
Partners Age (years)	30 (27, 34.25)	31 (27.5, 33)	0.63
BMI (kg/m2)	22.5 (20.1, 24.7)	20.6 (18.7 21.4)	0.45
Basal FSH (mIU/mL)	0.79 (0.26, 2.93)	1.55 (0.44, 3.58)	0.53
Basal E2 (pmol/L)	107.5 (73.4, 166.2)	106 (76.4, 126)	0.46
Basal LH (mIU/mL)	0.31 (0.10, 0.87)	0.35 (0.15, 1.5)	0.35
AMH (ng/ml)	2.12 (1.28, 3.69)	1.91 (1.53, 2.81)	0.06
AFC	8.5 (5, 12.75)	7.1 (4.5, 11.5)	0.028
Oocytes retrieved	11 (8.5, 13.5)	9 (6, 12.5)	< 0.001
FOI	1.15 (0.81, 1.18)	0.64. (0.34, 0.76)	< 0.001
Fertilized embryos	9 (7, 11)	7 (5.75, 12.5)	< 0.001
2PN embryos	7.5 (6, 9)	6 (4, 10)	0.031
Fertilization rate (%)			
IVF	85%	83%	0.54
ICSI	91%	92%	0.64
2PN rate (%)			
IVF	69%	69%	0.41
ICSI	83%	87%	0.33
Starting Gn doses (IU)	225 (150, 225)	225 (225, 300)	0.68
Stimulation days	14 (12.75, 16.25)	12.5 (11, 15)	0.54
Total Gn doses (IU)	3675 (2681.25, 4575)	3537.5 (2437.5, 4050)	0.12
E2 on HCG day (pmol/L)	6515 (4584.25, 10,633)	6219 (4087.5, 9078.5)	< 0.001
Endometrial thickness (mm)	10.2 (10.1, 12.5)	10 (10, 11.75)	0.49
Excellent quality embryos	6 (3, 7)	4 (3, 8)	< 0.001
Transferable embryos	3.5 (2, 8.25)	3 (2, 6.5)	0.022
Transferred embryos	2 (2, 2)	2 (2, 2)	0.98

The data are expressed by the median (25% quantile, 75% quantile), and the comparison between the two groups is performed by Mann Whitney test and Pearson Chi square test

*BMI* Body mass index, *FSH* Follicle stimulating hormone, *LH* Luteinizing hormone, *E2* Estradiol, *Gn* Gonadotropins, *AFC* Antral follicle count, *FOI* Follicle-to-oocyte index, *HCG* Human chorionic gonadotropin, *PN* Pronuclear

Logistic regression models were performed to investigate the effect of FOI on pregnancy in IHH cohort. In the univariate logistic regression model, each candidate factor affecting clinical pregnancy in IHH was examined end presented in Supplementary Table 2. The multivariate logistic regression analysis was performed using clinical pregnancy as the dependent variable and patients’ age, BMI, AMH, FOI and number of transferred embryos as independent variables. FOI was found to be the only significant independent factor affecting clinical pregnancy in IHH (OR = 1.131, *P* = 0.021) (Table 4).

**Table 4:** Multivariate logistic regression analysis for clinical pregnancy in IHH

**Dependent Variable**	**Variables**	**Odds ratio**	**95% confidence interval**	***P* value**
Clinical pregnancy	Age	1.104	0.983 to 1.551	0.12
	BMI	0.457	0.657 to 0.878	0.07
	AMH	0.532	0.476 to 1.251	0.21
	FOI	1.131	0.874 to 1.271	0.021
	Number of transferred embryos	1.011	0.691 to 1.042	0.92

*BMI* Body mass index, *AMH* Anti-Mullerian hormone, *FOI* Follicle-to-oocyte index

**Fig. 3:**
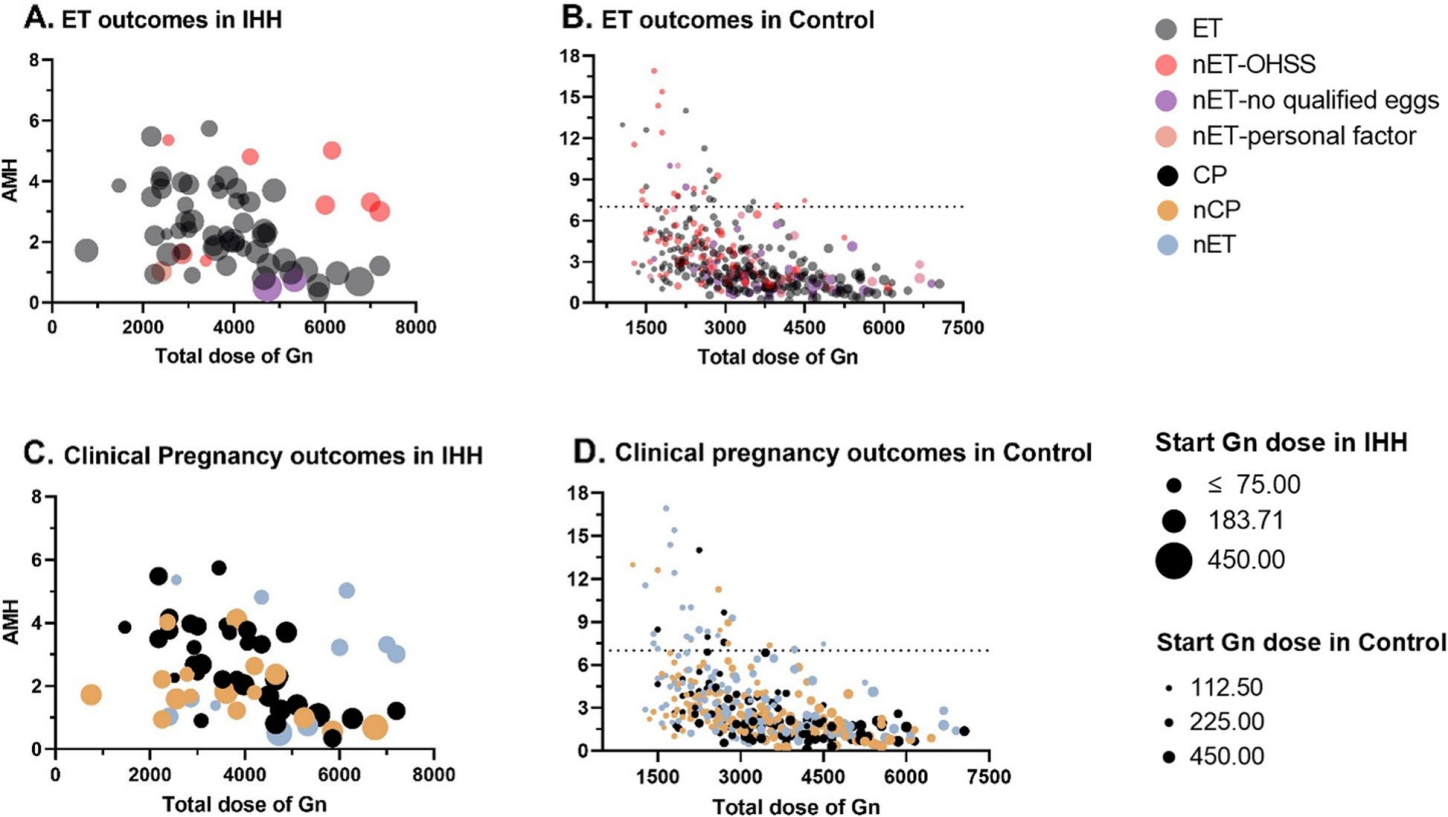
Comparison of ET outcomes and Clinical pregnancy outcomes in IHH and control. Bubble chart of total Gn doses, AMH, and starting Gn doses measured in IHH and control groups with the outcomes in embryo transfer (**A**, **B**) and clinical pregnancy (**C**, **D**). Grey bubbles represent patients who underwent ET; blue bubbles represent patients that didn't undergo ET; red, purple and pink represent cancellation for ET due to OHSS, no qualified eggs and personal factors; black represents clinical pregnancy after ET; yellow represents not clinical pregnancy after ET. Bubble size indicates the dose for starting Gn. The solid line represents the linear relationship between AMH and total Gn in IHH groups that underwent ET. ET: embryo transfer; nET: not embryo transfer; CP: clinical pregnancy; nCP: not clinical pregnancy; Gn: gonadotropin

Furthermore, a linear regression model for Gn usage in the cycle (including starting Gn, total Gn and stimulation duration) was performed based on the observation of the bubble plot of IHH patients (Fig. 3). As expected, the result showed that AMH was the significant factor for starting Gn and total Gn doses, reducing the dosage of Gn by 22.3 IU and 196.2 IU for each unit increase in AMH, respectively. Conversely, patients' age was found to have a significant positive effect on starting Gn and total Gn (Supplementary Table 3). In this analysis, AFC was the only factor correlated with the stimulation duration of Gn. Additionally, FOI showed a negative effect on starting Gn doses in low FOI group (*P* = 0.022).

## Discussion

This study utilized one of the largest cohorts of patients with IHH in China to investigate the ART outcomes in this population. The findings of this investigation demonstrated that patients with IHH exhibited positive responses to ovarian stimulation, resulting in successful outcomes for ART treatment. Furthermore, FOI proved to be an effective and novel marker for predicting ovarian response in IHH, especially in individuals with low ovarian response. Notably, a significant increase in the clinical pregnancy rate was observed from the low to normal FOI groups.

Consistent with prior research, this study also found that patients with IHH required a longer duration of gonadotropin stimulation and a higher total gonadotropin consumption compared to individuals with other infertility factors (e.g., male factor, tubal factor, and unexplained infertility) [12–15]. Such a long period of hypogonadotropic state may necessitate exogenous gonadotropin stimulation to activate the hypothalamic-pituitary-gonadal (HPG) axis from a “dormant phase” with normalization of steroidogenesis and gametogenesis [16, 17]. These results align with previous studies conducted by Yilmaz et al. and Ghaffari et al. [14, 15], which also investigated IVF outcomes in women with IHH. The former study examined 33 women with IHH and 47 women with male factor, whereas the latter study examined 81 women with IHH and 89 women with tubal factor. Both studies found that the retrieved oocytes and clinical pregnancy rate were similar between IHH groups and controls. Conversely, the present study found that the fertilization rate, 2PN rate, number of excellent quality embryos, and number of transferable embryos were higher in the IHH group. This discrepancy may be attributed to the Ghaffari et al. study's use of a higher total gonadotropin dose (average of 4845 IU) for ovarian stimulation in the IHH group, potentially compromising the quality of preimplantation embryos [14, 18]. In contrast, the average start gonadotropin dose and total gonadotropin dose for ovarian stimulation in IHH patients in the present study were 221 IU and 3390 IU, respectively, with 84.8% of cycles employing a start gonadotropin dose between 150–300 IU.

Effective fertility restoration in patients with IHH requires exogenous gonadotropins to regain reproductive function [16, 17]. Prediction of ovarian response to gonadotropins is crucial for achieving favorable IVF outcomes and avoiding super-ovulation-related complications. The current study is significant in several aspects, as it is the first to demonstrate the potential of FOI in predicting ovarian response in IHH patients and the correlation between FOI and clinical pregnancy in this population.

AMH and AFC, common markers of ovarian response, may not be suitable for IHH patients due to the difficulty of baseline measurement. The debate on whether serum AMH could serve as a prognostic marker of fertility in IHH still needs to be addressed [19, 20]. While previous research has suggested that AMH could be a promising marker of ovarian response in IHH patients undergoing IVF [21], emerging evidence indicates that AMH levels are not gonadotropin-independent throughout folliculogenesis [22, 23]. Conversely, FOI, an extension of the follicular output rate (FORT) concept [24], has been introduced as an alternative marker reflecting dynamic follicular development to ovarian stimulation [5]. FOI has been demonstrated to be effective in predicting ovarian sensitivity, particularly in patients with low response [5]. The previous study concluded that the low FOI (≤ 50%) indicates only a fraction of AFC exploited during ovarian stimulation [5]. Thus, the medication intervention for supplementation of FSH and LH may have the chance to improve the IVF prognosis. The present study’s findings support the use of FOI in predicting ovarian response to exogenous gonadotropins in IVF, especially in patients with low ovarian response based on the POSEIDON criteria.

This study represents the first attempt to investigate the predictive value of FOI in IVF outcomes in the IHH population. A few studies have yielded inconsistent results regarding association between FOI and reproductive outcomes. A retrospective study of 264 IVF cycles reported a positive correlation between increased FOI and live birth rate, which remained significant after age adjustment [25]. Conversely, Carosso et al. reported that FOI was not predictive of live birth in women with unexplained infertility and advanced reproductive age (≥ 39 years) in 740 IVF cycles [26]. In another study that analyzed 4323 fresh embryo transfer cycles, FOI was found to have a significant independent and linear association with clinical pregnancy [27]. It is possible that age-related oxidative stress and apoptosis may impact the AFC value and thus lead to changes in FOI [28]. In the present study, a comparison of pregnant and non-pregnant women in the IHH cohort revealed that FOI, number of retrieved oocytes, number of fertilized embryos, number of 2PN embryos, and number of excellent quality embryos were significantly higher in the pregnant group. This study found that the FOI was an independent predictor of clinical pregnancy in women with IHH undergoing IVF/ICSI cycles.

The present study benefits from a large IHH cohort, enabling the investigation of population characteristics in IVF procedure, such as the ovarian responsiveness, predictive value of FOI and clinical pregnancy outcomes. Given the unique nature of IHH, the study hypothesized that FOI would provide a better reflection of ovarian responsiveness to Gn stimulation compared to other traditional markers. Notably, this study is the first to demonstrate a correlation between FOI and clinical pregnancy in the IHH population. Nonetheless, certain limitations must be addressed. Firstly, the potential biases inherent in discrepancies between patients' data from different cycles were a concern, and only the first fresh cycle was analyzed in this study. Secondly, despite the large IHH cohort, stratified analyses were not possible due to sample size limitations. Larger sample size studies are needed to provide comprehensive information for physicians. Additionally, the study did not discuss other similar and novel parameters, such as FORT and ovarian sensitivity index (OSI), due to insufficient data. The following research will explore the varying predictive performance of FORT, OSI and FOI in IHH patients.

## Conclusion

Patients with IHH were good responders to ovarian stimulation. In women with IHH, FOI was well-performed predictor for ovarian response. Furthermore, FOI was an independent factor affecting clinical pregnancy in IHH undergoing IVF/ICSI.

## Supplementary Information

**Additional file 1: Supplementary Table 1.** ROC curves of selected ovarian reservation related markers in COS cycles to predict high and low ovarian response in IHH patients. **Supplementary Table 2.** Univariate logistic regression analyse of clinical pregnancy in IHH group. **Supplementary Table 3.** Linear regression model for the usage of Gn in IHH patients who underwent ET.

## Data Availability

The raw data supporting this study can be requested from the corresponding author.
